# The Podophage PM16 Enhances the Humoral Immune Response Against *Proteus mirabilis*

**DOI:** 10.3390/v18060669

**Published:** 2026-06-12

**Authors:** Lina Al Allaf, Anton V. Chechushkov, Vera V. Morozova, Yulia N. Kozlova, Tatiana A. Ushakova, Nina V. Tikunova

**Affiliations:** Laboratory of Molecular Microbiology, Institute of Chemical Biology and Fundamental Medicine Siberian Branch of Russian Academy of Sciences, 630090 Novosibirsk, Russia; morozova@1bio.ru (V.V.M.); ushakova@1bio.ru (T.A.U.)

**Keywords:** phage therapy, *Proteus mirabilis*, humoral immunity, PM16, bacteriophage, antibiotic resistance

## Abstract

Considering the therapeutic potential of the *Proteus mirabilis* PM16 podophage, the interaction between PM16, its host strain, and the mouse immune system was investigated. We evaluated how pre-existing humoral immunity to PM16 influences the immune response against *P. mirabilis* and the neutralization of the phage itself. Balb/c mice were divided into three groups and immunized two times with (1) 0.9% NaCl, (2) adjuvants, or (3) a mixture of PM16 and an adjuvant. Then, each group was subdivided into three subgroups: mock infection, infection with *P. mirabilis*, and infection with *P. mirabilis* followed by model phage therapy with PM16. The obtained results demonstrated that pre-immunization with PM16 enhanced the anti-*P. mirabilis* IgG antibody response upon bacterial challenge, indicating that the phage potentiates antibacterial immunity. In addition, pre-immunization elicited a significant anti-PM16 antibody response that resulted in in vitro neutralization of phage lytic activity. However, phage-neutralizing antibodies neither decreased the efficacy of phage therapy nor influenced bacteria-specific immune response. Thus, while PM16 can boost the host’s immune response against its bacterial host, the resulting humoral immunity also drives phage clearance through both direct and bacteria-mediated neutralization pathways, revealing a complex immunopharmacological relationship central to phage therapy.

## 1. Introduction

*Proteus mirabilis* is a Gram-negative bacterium characterized by swarming motility and the ability to produce urease [1]. This species has gained a lot of attention as an infectious agent that can cause various types of urinary tract infections (UTIs), including cystitis, pyelonephritis, and some cases of bacteriuria [2]. These infections sometimes develop into bacteremia and life-threatening urosepsis, in addition to the formation of urinary stones [2,3,4]. Biofilm formation in catheters is another serious condition caused by *P. mirabilis* that usually leads to catheter blockage and infected urine reflux, threatening the lives of many catheterized patients [5].

Regarding the immune response against *P. mirabilis*, in vivo and in vitro studies showed that *P. mirabilis* flagellin can induce innate immunity, activating the expression of pro-inflammatory chemokines in UTI animal models [6,7]. Furthermore, *P. mirabilis* flagellin is able to attract leukocytes to the site of infection, which leads to an inflammatory response. However, this response does not help in eliminating the established infection [8]. It is believed that antigen presentation occurs in UTIs, leading to specific T- or B-cell activation. The detailed mechanism underlying this process is still unknown [9]. *P. mirabilis* uses different strategies to avoid the host’s immune response. It escapes the antibody response by producing a modified type of flagellin or secreting proteases that affect IgA antibodies [10,11]. Additionally, capsular polysaccharides (CPSs) and some types of O-antigens were described as factors that help to develop the bacterial infection and evade the host immune response [4,12].

*Proteus mirabilis* can be naturally resistant to several antibiotics, including polymyxins (colistin), nitrofurans, tigecycline, and tetracycline [13]. Multiple resistance genes have been reported in *P. mirabilis* isolates, including genes encoding narrow-spectrum β-lactamases, inhibitor-resistant TEM β-lactamases, acquired cephalosporinases, and carbapenemases. Moreover, recent studies indicated the emergence of strains with multidrug resistance to antibiotics used to treat *P. mirabilis* UTIs and catheter-associated UTIs (CAUTIs) [14,15,16,17,18,19,20,21]. Phage therapy has recently been considered a possible opportunity to treat antibiotic-resistant infections, including those caused by *P. mirabilis* [5]. Using a cocktail of phages (the podovirus vB_PmiP_5460 and myovirus vB_PmiM_5461) to coat catheters, a notable decrease in biofilm formation by *P. mirabilis* has been shown [22]. Another study described the use of a cocktail of *P. mirabilis* podophages, ΦRS1-PmA, ΦRS1-PmB, and ΦRS3-PmA, on models that mimic the conditions where *P. mirabilis* can cause biofilm formation in catheters [23].

The use of phages in therapy has raised many questions about the safety and efficacy of this approach. The production of phage-specific antibodies, mainly IgM and IgG subclasses, has been reported [24,25], accompanied by the secretion of IgA into the bloodstream and mucosal surfaces [24]. Interestingly, these antibodies were detected before the start of immunization courses, which may be due to the fact that contact with phages from the microbiota contributes to the formation of pre-existing antibodies [24]. The induction of a humoral immune response appears to depend on the phage dose and the route of administration [26]. Studies have shown that the presence of phage-neutralizing antibodies in the blood depends on the specific phage, the immunogenicity of phage proteins, and sometimes on the redundancy of target proteins [25,26,27]. Notably, the meta-analysis data demonstrated that phage neutralization does not necessarily lead to the failure of phage therapy, whereas phage-specific non-neutralizing antibodies can reduce phage titers without direct neutralization [28]. The downside is that sufficient time is required for antibodies to reach neutralizing levels, even in previously immunized subjects. Therefore, the study of phage-specific antibodies should be conducted in the context of broader questions.

Previously, it has been demonstrated that the *Proteus* phage PM16 can induce a transient increase in blood levels of cytokines (TNF, IFN-γ, IL-1β, and Il-6) in CD-1 mice after triple immunization without adjuvants, but did not elicit IgM and IgG anti-phage antibody responses [25]. In addition, PM16, being administered with adjuvants, can both effectively control subcutaneous *P. mirabilis* infection in mice and induce long-term specific humoral immunity against subsequent reinfection via macrophage priming [29]. We have also shown an increase in the level of phage-specific antibodies; however, their phage-neutralizing ability was not investigated [30].

Given its therapeutic potential, we designed a new experiment involving pre-immunization of mouse models with PM16. The aim was to determine whether specific antibodies to the phage could interfere with bacterial-specific humoral immunity. In this study, we examined the interaction between the *Proteus* phage PM16, its host strain *P. mirabilis*, and the animal immune system. Here, we describe the effect of pre-immunization with podophage PM16 on the humoral immune response against *P. mirabilis* and the neutralization of the phage itself.

## 2. Materials and Methods

### 2.1. Animals

Two-month-old male Balb/c mice were obtained from the animal care facility in the State Research Center of Virology and Biotechnology VECTOR, Novosibirsk. Animals were housed under a normal light–dark cycle; water and food were provided ad libitum. All animal experiments adhered to the guidelines for the protection of animals in scientific research as set out in EU Directive 2010/63/EU. All animal studies received approval from the Inter-institutional Bioethics Committee at the Institute of Cytology and Genetics, SB RAS, Novosibirsk, Russia.

### 2.2. Bacteriophage PM16 and Its Host

Bacterial strain *P. mirabilis* CEMTC 73 was obtained from the Collection of Extremophilic Microorganisms and Type Cultures (CEMTC) of the Institute of Chemical Biology and Fundamental Medicine (ICBFM), SB RAS, and cultivated in Lysogeny Broth (LB). PM16 propagation was conducted by infecting *P. mirabilis* CEMTC 73 culture (OD600 = 0.6) at a multiplicity of infection (MOI) of 0.1. Cultures were then incubated at 37 °C with shaking until the beginning of bacterial lysis. Phage particles were precipitated using a mixture of polyethylene glycol 8000 (AppliChem, Darmstadt, Germany) with 2.5 M sodium chloride, and then resuspended in phosphate-buffered saline (PBS), pH 8.0. The phage suspension was then purified by centrifugation (22,000 rpm, 2 h, 4 °C) in a cesium chloride gradient [31]. The purified phage was dialyzed against PBS, and the titer was determined to be 5 × 10^11^ plaque-forming units per mL (PFU/mL).

The endotoxin level of the purified preparation of PM16 was then measured. A serial dilution ranging from 10^6^ to 10^12^ PFU/mL was prepared in sterile 0.9% NaCl and used with a Limulus amebocyte lysate (LAL) assay (Charles River Laboratories Inc., Charleston, SC, USA) according to the manufacturer’s instructions. A concentration of 10^8^ PFU/mL corresponded to an endotoxin level of 0.5 EU/mL, which falls within the acceptable range for pharmaceutical preparations intended for parenteral administration.

### 2.3. Preparing the Microscopic Plates Coated with P. mirabilis

A sterile solution of poly-L-lysine (0.1%) was added to each well of a specialized microscopic 96-well mu-plate (ibidi, Gräfelfing, Germany) and exposed to 37 °C for 1 h. The solution was then removed, and the wells were washed three times with sterile distilled water. A suspension of *P. mirabilis* CEMTC 73 in PBS (10^8^ colony-forming units (CFU) per well) was added to the coated poly-L-lysine plate and incubated at 37 °C for 30 min. Next, the suspension was removed and the unattached bacteria were washed three times. To assess the optimal bacterial density for subsequent analysis, the wells were scanned with an LSM710 confocal microscope at 1000× magnification (Carl Zeiss, Oberkochen, Germany), followed by the detection of individual bacterial cells using the CellProfiler v.4.2.4.

### 2.4. Study of Serological Response to P. mirabilis

The serological response to *P. mirabilis* was evaluated using confocal microscopy as described previously [29]. Briefly, bacterial cells were incubated with sera dilutions (1/500) and then fluorescently screened using anti-IgG Alexa Fluor 488-conjugated donkey anti-mouse antibodies (Invitrogen, Waltham, MA, USA) and intercalating stain Hoechst 33342 (Life Technologies, Carlsbad, CA, USA). All images were obtained using identical microscopic settings and then processed using CellProfiler software. The mean fluorescence intensity (MFI) of the identified bacterial cells was calculated.

### 2.5. ELISA of Mice Sera

Determining anti-PM16 antibodies in mouse sera was performed using ELISA. Wells of 96-well plates (Medpolymer, Saint Petersburg, Russia) were coated with PM16 (10^8^ PFU/well) in PBS. After washing, wells were blocked using a solution of 5% skimmed milk in PBS with 0.1% Tween-20. Five-fold serial dilutions of sera were prepared, starting from a 1:100 dilution, and then added to the wells and incubated at 37 °C for 2 h. Washing was followed by adding anti-mouse IgG peroxidase-conjugated rabbit polyclonal antibodies (Biosan, Novosibirsk, Russia) and plates were incubated at 37 °C for 1 h. Wells were then stained with 3,3′,5,5′-tetramethylbenzidine (Amresco, Solton, OH, USA).

### 2.6. Analysis of Neutralization of Bacteriophage Lytic Activity by Mouse Sera

Aliquots (20 μL in PBS) of serial dilutions of podophage PM16 were mixed with equal volumes of anti-phage pooled serum samples and incubated at room temperature for 1 h. Then, each mixture was dropped onto a fresh layer of *P. mirabilis* in the top agar and plates were incubated at 37 °C overnight to reveal phage plaques. Phages mixed with non-immune mouse serum and phages mixed with PBS were used as controls. All experiments were done twice, in two technical repeats. Neutralization titer was calculated according to N = (V_0_ − Vn)/V_0_ × 100%, where V_0_ is the number of plaques of phage samples incubated with PBS and Vn is the number of plaques of phage samples incubated with mouse sera.

### 2.7. PM16 Labeling with AF488 Fluorescent Dye

Phage PM16 was fluorescently labeled using Alexa Fluor 488 NHS-ester (Sigma-Aldrich, St. Louis, MO, USA). Phage suspensions at a concentration of 10^10^ PFU/mL were mixed with equal volumes of AF488 solutions prepared at dye concentrations of 1, 10, or 100 µM. The NHS-ester group of the dye reacts covalently with primary amine groups on the phage capsid proteins to form a stable amide bond. The mixtures were incubated at 4 °C for 24 h to facilitate this conjugation reaction. To remove unreacted free dye, the stained and control (unstained) phage suspensions were purified using size-exclusion chromatography on Sephadex G-50 resin (Merck KGaA, Darmstadt, Germany). The lytic activity of the labeled and purified PM16 was confirmed using a plaque assay to ensure that the labeling process did not significantly impair infectivity. Aliquots of the solutions were added to a fresh layer of *P. mirabilis* host on top agar, and plaques were counted after overnight incubation.

### 2.8. Study of Fluorescently Labeled PM16 Phage Binding to P. mirabilis

*P. mirabilis* cells immobilized on the surface of a 96-well mu-plate (ibidi, Gräfelfing, Germany) were opsonized with the sera of experimental animals for 2 h at 37 °C. After washing, the wells were blocked with a 5% bovine serum albumin solution (for 1 h at 37 °C) to prevent immobilization of phages on the well surface. Fluorescently labeled PM16 phages (10^9^ PFU/well) were added to the wells and incubated for 30 min at 37 °C. The wells were then washed and additionally stained with anti-IgG antibodies, which made it possible to control the absence of residual phage-neutralizing antibodies in the wells.

Fluorescence intensity (MFI) was measured for approximately 1000 individual bacterial cells per technical replicate (slide). To obtain a robust measure of central tendency for each slide while minimizing the influence of skewed cell-level distributions, the mean MFI was calculated per slide. For each mouse (*n* = 3 per group), the three slide medians were then averaged to yield a single biological replicate value (mouse-level MFI). These mouse-level values were used for conventional group comparisons.

To fully leverage the hierarchical structure of the data and account for variability at the mouse and slide levels, a linear mixed-effects model was fitted to the log-transformed raw cell MFI values [log(MFI + 1)]. The model included the experimental group as a fixed effect, with nested random intercepts for mouse and for slide within each mouse. Model fitting was performed using Python’s statsmodels library. Pairwise comparisons among groups were conducted using Tukey’s honestly significant difference (HSD) test on the estimated marginal means derived from the model, with family-wise error rate controlled at *p*-val < 0.05.

Conventional analysis of aggregated mouse-level data was performed with a non-parametric Kruskal–Wallis test, which was further used with post hoc pairwise comparisons carried out using Dunn’s test and Bonferroni correction for multiple comparisons. All tests were two-sided with significance set at *p* < 0.05.

### 2.9. Analysis of Phage Epitope Conservation

To investigate the potential for immunological cross-reactivity between *Proteus* phage and bacterial proteins, we performed a comparative analysis of PM16 structural protein conservation and their predicted B-cell epitopes. Amino acid sequences for five major structural proteins of podophage PM16 (GenBank: NC_027342.1) were used as queries: gp31 (major capsid protein, YP_009147866.1), gp34 (tail tubular protein, YP_009147868.1), gp37 (internal core protein, YP_009147871.1), gp38 (tail fiber protein, YP_009147872.1), and gp45 (spike protein, YP_009147879.1). Protein–protein BLAST (PSI-BLAST) (https://blast.ncbi.nlm.nih.gov) [32] was performed against the NCBI non-redundant protein database (nr) using an E-value cutoff of 1 × 10^−9^ to identify distant similar sequences. The resulting hit sequences were filtered to retain only those belonging to the genus *Proteus* or identified as phage-related.

Filtered similar sequences for each PM16 structural protein were aligned using Clustal Omega (v1.2.4) [33] with default parameters. To quantitatively assess sequence conservation at each alignment position, we calculated the Shannon entropy (H) using the equation H(i) = −Σ (p(X,i) × log_2_ p(X,i)), where p(X,i) is the frequency of amino acid ‘X’ at position ‘i’. A normalized conservation score (C) was derived as C(I) = 1 − H(I)/log_2_(Hmax), with Hmax = 20 for proteins. Scores close to 1 indicate perfect conservation across homologs.

B-cell epitopes were predicted from the reference PM16 protein sequence using the BepiPred-3.0 algorithm [34] using the default combined model (CNN + LSTM), and residues with a score above the recommended threshold of 0.15 were designated as potential epitope residues. The multiple sequence alignment, conservation scores, and B-cell epitope prediction tracks were integrated and visualized programmatically using Python 3.11 to generate composite plots, aligning the three data types (alignment, entropy, epitope score) by residue position.

### 2.10. Analysis of Protein Functional Similarity and 3D Structure Mapping and Modeling

HHPred-based [35] analysis was performed for the PM16 gp45 protein to evaluate the functional properties of regions with predicted epitopes. The following search parameters were set up: MSA generation method—Hhblits → UniRef30, E-value cutoff 1 × 10^−3^, MSA generation iterations—3.

The PM16 gp45 protein 3D model was predicted with alpha-fold [36]. Overall scores for predicted models were thresholded by 0.85. The model *.cif files were utilized for the following 3D visualization and mapping of predicted epitopes with Python 3.12 and relevant packages. In brief, the amino acid model was parsed with Bio.PDB [37]. Bepipred3-predicted epitopes were mapped on the structure with the py3Dmol package (https://github.com/avirshup/py3dmol, accessed on 23 December 2026).

## 3. Results

### 3.1. Study Design

**Figure 1:**
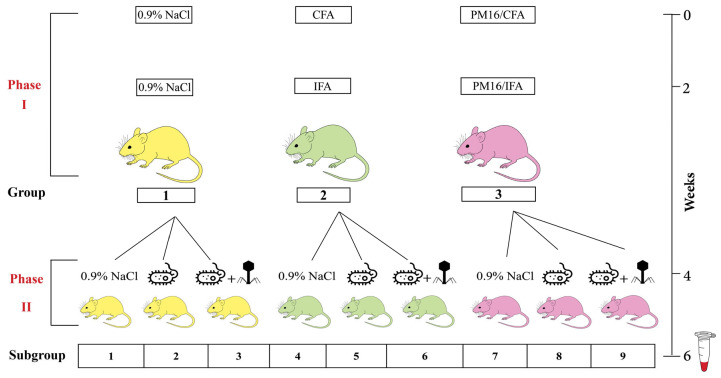
Study design. Mice were immunized twice (Days 0 and 14) and challenged (Day 28) with the indicated treatments. All injections were administered intraperitoneally. Group sizes were *n* = 9 for immunization and *n* = 3 for challenge subgroups. CFA, complete Freund’s adjuvant; IFA, incomplete Freund’s adjuvant.

The experimental procedures consisted of two phases (Figure 1). In Phase I, mice were divided into three groups (*n* = 9, each). Animals from the first group were intraperitoneally injected with a sterile 0,9% NaCl solution, the second group was injected with complete Freund’s adjuvant (CFA) without PM16, and the third group was immunized with PM16 phage (10^9^ PFU per mouse) in the presence of CFA. No group immunized with only PM16 phage was present, as PM16 has been previously shown to be non-immunogenic per se [25]. Two weeks later, mice were re-immunized the same way, except that incomplete Freund’s adjuvant (IFA) was applied instead of CFA.

In Phase II, four weeks after initial immunization, mice from each group were subdivided into three subgroups (*n* = 3, each): mock infection, infection with *P. mirabilis*, and infection with *P. mirabilis* followed by model phage therapy with PM16 (10^9^ PFU per mouse) after 1 h of the bacterial infection (Figure 1). Mock infection was provided with sterile 0.9% NaCl solution. *P. mirabilis* was introduced at 10^8^ CFU per mouse. All infection procedures were performed intraperitoneally.

Blood samples were collected two weeks after the Phase II initiation (Figure 1). Individual serum samples from each mouse were used for immunological experiments; for the PM16 neutralization experiment, serum samples were pooled within each subgroup.

### 3.2. Serological Response to P. mirabilis

**Figure 2:**
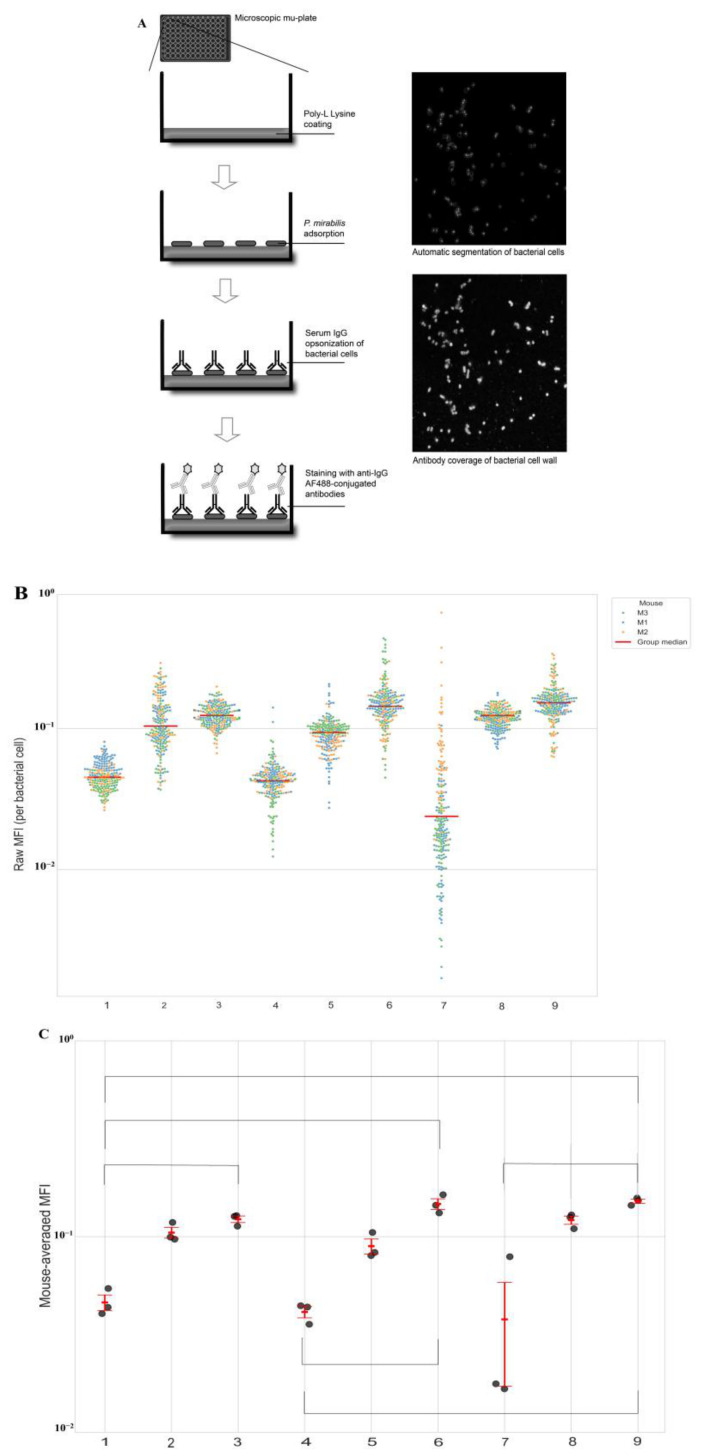
*P. mirabilis* binding by serum antibodies. (**A**) Bacteria were adsorbed on a microscopic 96-well mu-plate and opsonized with mouse sera from each subgroup, followed by staining with AF488-conjugated anti-IgG antibodies and Hoechst dye. Wells were imaged using an LSM710 confocal microscope at 1000× magnification. The CellProfiler software was used for segmentation and measuring of images. (**B**) The results of quantitative analysis representing individual bacterial cells from each mouse (shown with colors). (**C**) Per-mouse mean +/− SEM values for each group. The Kruskal–Wallis test (with Dunn’s post hoc) was applied for statistical analysis of the acquired data (each square bracket indicates *p*-val < 0.01). MFI—mean fluorescence intensity.

To assess how PM16 administration and pre-immunization influence the humoral response against the bacterial host, we measured serum IgG binding to *P. mirabilis* cells using a fluorescence-based high-throughput assay (Figure 2A; see Supplementary Material for validation). A linear mixed-effects model was fitted to log-transformed raw fluorescence values, with the experimental group as a fixed factor and the mouse and technical slide as nested random effects; complete model outputs are provided in Table 1. Mice that received only a bacterial challenge (subgroups 2, 5, and 8) developed a moderate but detectable anti-*P. mirabilis* IgG response. When PM16 was administered 1 h after infection (phage therapy model, subgroups 3, 6, and 9), IgG binding increased significantly across all three Phase-I backgrounds—saline, adjuvant alone, and PM16 plus adjuvant—with each pairwise comparison between infection-alone and infection-plus-phage subgroups yielding Tukey-adjusted *p* values < 0.001. Within the infection-only Phase-II subgroups, animals pre-immunized with PM16 plus adjuvant (subgroup 8) showed stronger IgG binding than those that received only saline (subgroup 2) or adjuvant alone (subgroup 5), indicating that prior phage exposure primes the host for a more robust antibacterial response. Remarkably, the combination of PM16 pre-immunization and subsequent phage therapy (subgroup 9) produced an IgG level statistically indistinguishable from that of the strongest adjuvant-based therapy group (subgroup 6; adjusted *p* = 0.071), suggesting that pre-existing anti-phage immunity can augment the antibacterial humoral response to a degree comparable to a potent adjuvant-driven regimen. As expected, all mock-infected subgroups (1, 4, and 7) displayed the lowest IgG binding. Analysis of mouse-level aggregated mean fluorescence intensities (Figure 2C) confirmed the separation of the high-binding subgroups 6 and 9 from the mock-infected controls (Kruskal–Wallis *p* = 0.0022, Dunn’s post hoc *p* < 0.05). Collectively, these results demonstrate that PM16 administration consistently enhances the anti-*P. mirabilis* IgG response. Pre-immunization did not diminish this enhancement; indeed, the highest IgG levels were observed in animals that received both prior PM16 exposure and subsequent phage therapy, indicating that phage-specific immunity does not impair the development of antibacterial humoral immunity.

### 3.3. Serological Response to PM16

**Figure 3:**
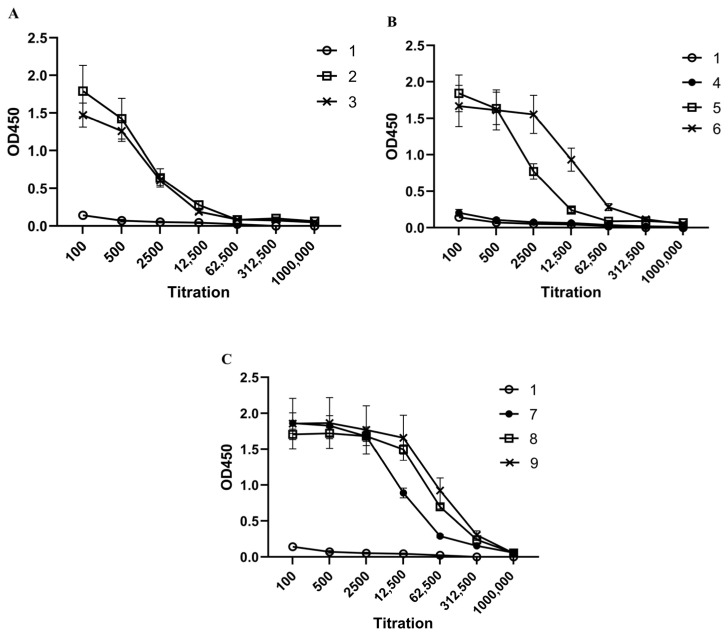
ELISA evaluation of serum binding to PM16. Charts (**A**–**C**) represent a comparison of different subgroup sets. The median of the obtained results was used to perform curve fitting and relative binding activity assessment.

To assess serum anti-PM16 IgG in each subgroup, an analysis of antibody binding to the phage by ELISA was performed at the end of Phase II. The ELISA results indicated that in non-immunized mice (0.9% NaCl in Phase I), PM16 binding antibodies were observed (Figure 3A) if the animals were infected with *P. mirabilis* (subgroup 2) or infected with *P. mirabilis* with a subsequent PM16 phage therapy model (subgroup 3). In subgroups of animals that received only adjuvants in Phase I (Figure 3B), a similar but more pronounced effect was observed; in this case, infection followed by phage therapy was accompanied by an increase in serological response (subgroup 6) compared with infection without phage therapy (subgroup 5). Animals pre-immunized with PM16 + adjuvants in Phase I (Figure 3C) demonstrated high levels of IgG (subgroup 7). Introduction of *P. mirabilis*, as well as infection with *P. mirabilis* followed by phage therapy with PM16, enhanced the serological response (subgroups 8 and 9 versus subgroups 5 and 6, respectively).

### 3.4. Phage Neutralization

**Figure 4:**
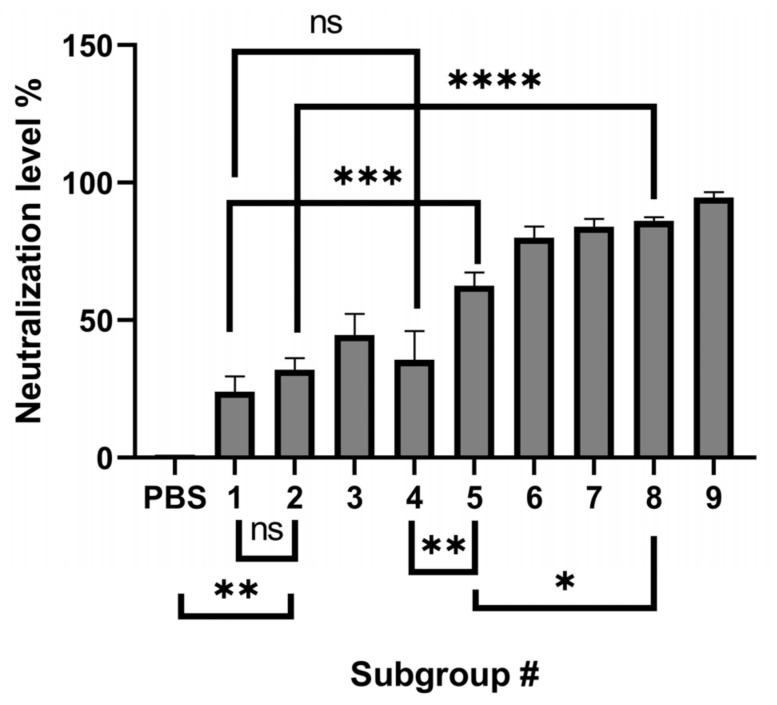
PM16 neutralization with mouse serum samples. PM16 was incubated with experimental sera followed by titration on *P. mirabilis* bacterial lawn on Petri dishes. The number of lytic plaques was counted 18 h after the application. Sera from mice of matching subgroups were pooled. Each point represents mean ± SD of three replicates of the phage-neutralization assay. Neutralization level was calculated according to N = (V_0_ − Vn)/V_0_ × 100%, where V_0_ is the number of plaques of phage samples incubated with PBS and Vn is the number of plaques of phage samples incubated with mouse sera. Kruskal–Wallis analysis was applied for statistical inference of the acquired data (* *p* < 0.05, ** *p* < 0.01, *** *p* < 0.001, **** *p* < 0.0001).

To estimate phage-neutralizing activity of mouse sera, PM16 phage particles opsonized with inactivated sera from each subgroup were tested for lytic activity (Figure 4). Relative to the NaCl-injected control (subgroup 1), no significant neutralization was detected in subgroups 2–4. Subgroups 5 and 6 exhibited significantly increased neutralizing activity (*p* = 0.0007 and *p* < 0.0001, respectively). Notably, mice that received the host bacterium alone (subgroup 5) showed neutralizing IgG antibodies compared to subgroup 4 (*p* = 0.01), an effect also observed in subgroup 6 (*p* = 0.0002 vs. subgroup 4), with no difference between subgroups 5 and 6. Mice immunized with PM16 plus adjuvants (subgroups 7–9) displayed the most enhanced neutralization (*p* < 0.0001 vs. subgroup 1), but no significant difference was detected between subgroup 6 and subgroups 7–9. This lack of significant difference cannot be attributed to assay saturation, because at PM16, the highest concentration (10^5^ PFU/μL), the neutralization values (∼80% for subgroup 6) were not at the detection limit, and the assay successfully resolved lower activity in subgroup 5.

### 3.5. Serum-Mediated Prevention of PM16 Adsorption on P. mirabilis Cells

**Figure 5:**
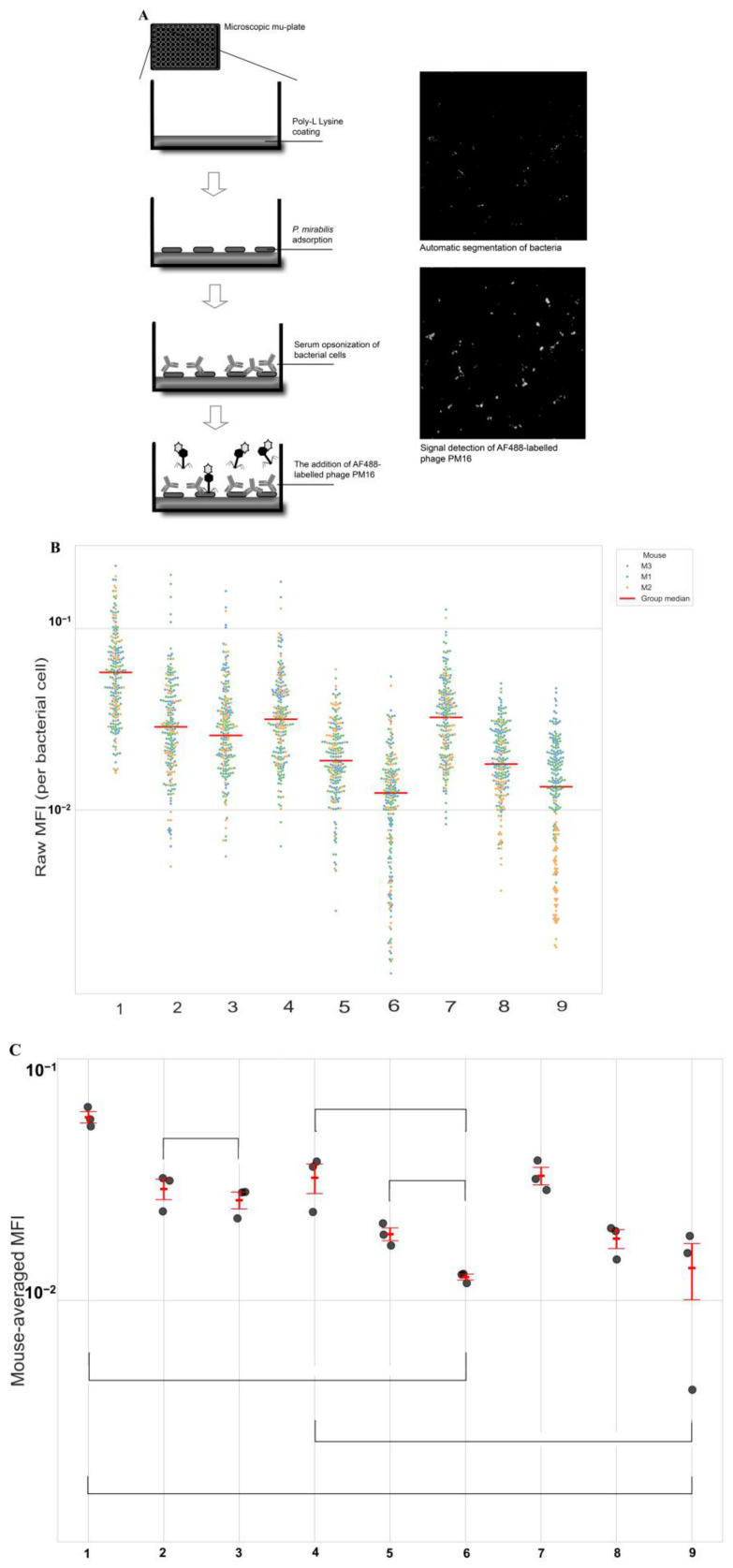
(**A**) PM16 binding to *P. mirabilis* cells opsonized with experimental sera. After serum opsonization of the bacteria, a wash procedure was performed to remove traces of antibodies not bound to bacterial cell walls. After that, AF488-labeled PM16 was introduced into plate wells and incubated for 30 min. (**B**) The results of quantitative analysis representing individual bacterial cells from each mouse (shown with colors). (**C**) Per-mouse mean +/− SEM values for each group. Kruskal–Wallis test (with Dunn’s post hoc) was applied for statistical analysis of the acquired data (each square bracket indicates *p*-val < 0.01).

To test whether serum antibodies were capable of blocking the initial step of phage infection—attachment to the host cell—we designed an in vitro binding assay. *P*. *mirabilis* cells adsorbed onto microplates were first opsonized with sera from each experimental subgroup. After rigorous washing to remove unbound antibodies, fluorescently labeled PM16 was added and the residual phage binding to individual bacteria was quantified by confocal microscopy (Figure 5A). Control experiments confirmed that the final wash fluid lacked detectable phage-neutralizing activity, ensuring that any inhibition of binding was due to antibodies retained on the bacterial surface and not carryover of free antibodies.

A linear mixed-effects model applied to the log-transformed cell-level fluorescence data revealed a significant overall effect of the experimental condition (likelihood ratio test, *p* < 0.001; full model output in Table 2). Relative to the non-opsonized control (subgroup 1), serum from every immune subgroup (subgroups 2–9) significantly reduced PM16 binding, with decreases in mean fluorescence ranging from 2.7% to 4.9% on the original scale (all Tukey-adjusted *p* < 0.001).

Unexpectedly, substantial inhibition was already observed with sera from mice that had received only *P. mirabilis* infection, without any exposure to PM16 (subgroups 2, 5). This indicates that antibacterial antibodies produced during infection can block phage attachment, most likely by occupying the same surface receptors that PM16 uses for adsorption.

When PM16 was administered 1 h after infection in the phage therapy model (subgroups 3, 6, and 9), phage binding was reduced slightly but statistically significantly further compared with the corresponding infection-only subgroups (2, 5, and 8; all Tukey-adjusted *p* < 0.001). However, the magnitude of this additional reduction was very small (mean differences on the log scale all below 0.01). At the mouse-aggregated level, these small differences were not significant for subgroups 5 versus 6 (Dunn’s test, *p* = 0.33) nor for subgroups 8 versus 9 (*p* = 0.57), and the two therapeutic phage administration groups—subgroup 6 (adjuvant background plus phage) and subgroup 9 (PM16 pre-immunization plus phage)—showed indistinguishable levels of binding inhibition (Tukey *p* = 0.99).

Together, these results demonstrate that the observed in vitro neutralization of PM16 can proceed through two complementary mechanisms. First, receptor blockade by antibacterial antibodies that occupy phage attachment sites on the host cell. Second, direct phage neutralization by phage-specific antibodies that bind to the phage particle itself. The strong blocking activity of sera from bacteria-only exposed animals and the marginal additional effect of phage co-administration suggest that receptor blockade is the predominant mechanism of attachment inhibition in this model. 

### 3.6. Analysis of PM16 Protein Similarity with Proteins Encoded by the P. mirabilis Genomes

To address the question of why sera from bacteria-only exposed animals (subgroups 2 and 5) demonstrated cross-reactivity with PM16 phage (as is evident from the ELISA results, Figure 3), it was assumed that bacteria could produce proteins that expose epitopes resembling those exhibited on PM16 particles. The following PM16 structural proteins were selected: gp31 (capsid protein), gp34 (tail tubular protein), gp37 (internal core protein), gp38 (tail fiber protein), and gp45 (spike protein). A search for proteins similar to PM16 structural proteins [30] was performed in the non-redundant protein database (NCBI) with the PSI-blast algorithm (E-value cutoff 1 × 10^−9^). For each protein, the output data was filtered for those similar proteins that belong to *Proteus*-specific phages [38]. As a result, a set of highly conserved similar proteins was obtained, and their sequences were searched for potential B-cell epitopes using the Bepipred3 algorithm.

**Figure 6:**
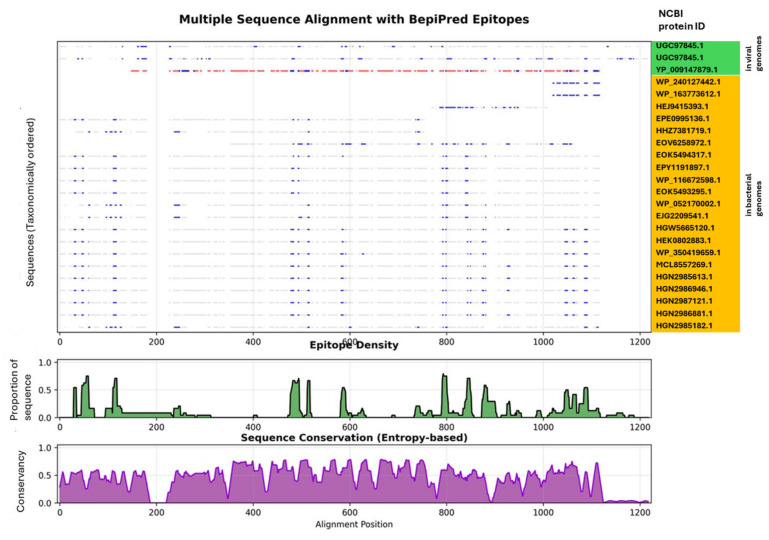
Analysis of B-cell epitope potential and sequence conservation in the PM16 gp45 spike protein. Multiple sequence alignment of the PM16 gp45 protein with similar sequences from other *Proteus* phages and prophage proteins. Gray lines indicate aligned sequences for similar proteins (white gaps correspond to gaps in alignment). The red line indicates the sequence of the PM16 gp45 protein. Epitope density represents predicted B-cell epitope density across the protein sequence, calculated using the Bepipred 3.0 algorithm (threshold = 1.56). Peaks indicate regions with high epitope representation among aligned sequences. The sequence conservation plot demonstrates the Shannon entropy-based conservation score (calculated as 1–Shannon_entropy) for each amino acid position (averaged by a window of 10 amino acids). Higher values indicate higher sequence conservation across the sequences. Similar sequences were retrieved via PSI-BLAST (E-value cutoff: 1 × 10^−9^).

**Figure 7:**
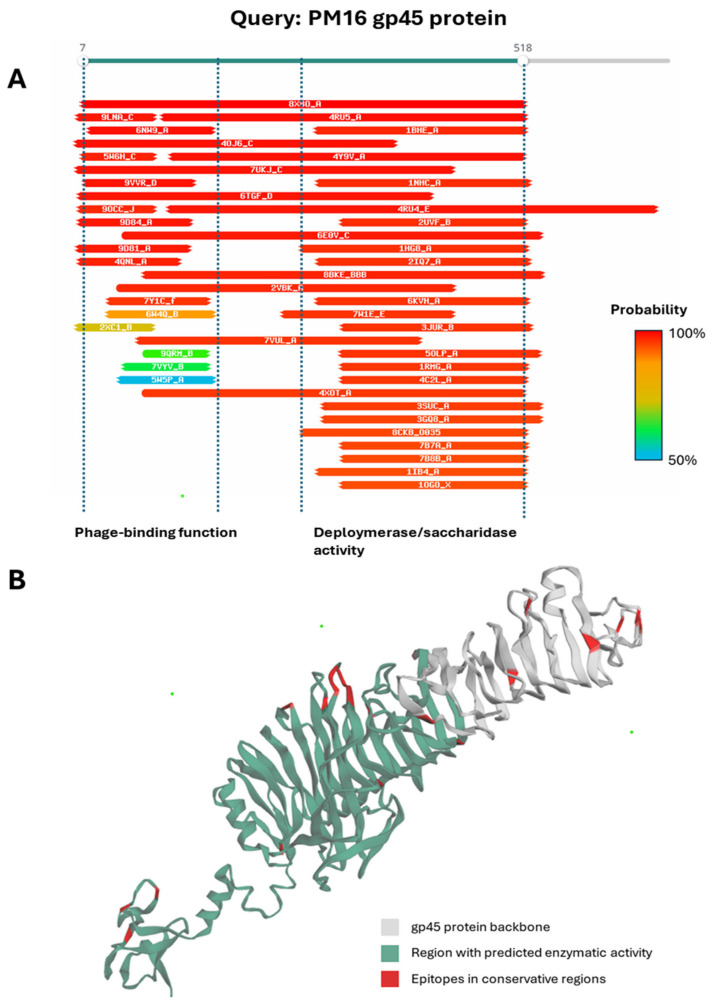
Mapping of the predicted epitopes on gp45 3D visualization of proteins. (**A**) The result of HHPred analysis of the PM16 gp45 protein revealed that it contains a long N-terminal region (shown in green) with similarity to functionally annotated spike proteins that provide carbohydrate-specific enzymatic activities. (**B**) 3D mapping of epitopes predicted for conserved regions shows that some of the epitopes may lie inside functionally significant regions.

Among the PM16 structural proteins, gp31, gp37, and gp45 contained a lot of sequences that were candidates for B-cell epitopes, whereas gp34 and gp38 were poor in potential B-cell epitopes. Interestingly, gp31 and gp37 showed high conservation scores compared to the proteins of *P. mirabilis* phages, and only gp45 (the spike protein) aligns to both *P. mirabilis* phage and a broad range of proteins encoded by the genomes of different *P. mirabilis* strains (Figure 6). Further analysis indicated that the genes encoding these *P. mirabilis* proteins were part of prophage sequences unrelated to PM16 or were randomly integrated into the genome with the potential ability to be expressed under constitutive or stress-induced promoters. This indicates that different *Proteus* strains could encode the highly conserved proteins in their genomes with potential candidates for B-cell epitopes. Additionally, HHpred analysis shows (Figure 7) that predicted epitopes cover the regions that are structurally similar to known functional motifs possessing bacterial cell wall binding and depolymerase activity. Neutralization of these motifs could explain the observed results of PM16 decreased binding and lack of activity.

Provided observations demonstrate, first, the high conservation among some structural proteins of bacteriophage. In addition, the gp45-encoding gene is widely present in different bacterial genomes. Second, the obtained results indicate that the conservative regions can share the potential B-cell epitopes in the gp45-like proteins. Taken together, this indicates that closely related phages possessing highly conserved plausibly immunogenic structures may provide cross-reactivity not only between themselves but also between phages and bacteria.

## 4. Discussion

Lytic *P. mirabilis* phage PM16 is a podovirus described as a stable phage with a short latency period and large burst size [30]. This phage is characterized by the occurrence of low phage resistance. Phage-resistant *P. mirabilis* obtained after PM16 infection revealed a non-swarming phenotype and the absence of flagella; therefore, the ability to spread infection was decreased in *Proteus* cells resistant to phage PM16 reinfection [30]. This makes PM16 a good choice to be included in a *Proteus* phage cocktail to control *Proteus* infection.

Our previous study demonstrated that PM16 therapy during an active *P. mirabilis* infection not only reduced bacterial burden in animals but also significantly enhanced the antibacterial IgG response, suggesting an immune-priming capacity distinct from its direct lytic action [34]. The current study was designed to further explore this immunopharmacological relationship, specifically to test the hypothesis that the induction of phage-neutralizing antibodies can be an intrinsic part of a beneficial immune response rather than a therapeutic obstacle to treatment.

In our experiments, we evaluated the possibility of PM16 pre-existing humoral immunity to influence the immune response against *P. mirabilis.* Additionally, we investigated the neutralizing ability of sera from PM16 pre-immunized mice in a phage therapy model. In this model, a non-lethal murine *P. mirabilis* infection was selected, which makes it possible to track changes in the immune status of animals after *P. mirabilis* infection and experimental PM16 phage therapy. PM16 phage was chosen to be used in the phage therapy model, which was previously shown to be non-immunogenic *per se* [25]. Since the used bacterial model is not accompanied by lethality or pronounced manifestations of a bacterial infection, the parameters of humoral immunity in relation to the host bacterium were used as a measure of effectiveness.

The results of the serological response to *P. mirabilis* indicated that previous immunization with PM16 can increase the immunological response against the bacterium. Based on this observation, we can suppose that PM16 can enhance the immune response against *P. mirabilis*. Moreover, injection with PM16 soon after infection with *P. mirabilis* (phage therapy model) increases the serological response to the bacteria, an effect that was additionally boosted both by mock (adjuvants only) and full immunization with PM16. These results demonstrated that phage-specific immune response is an important part of phage therapy, with phages potentially serving as mediators between targeted bacteria and the immune response.

Consistent with previous studies [24,31,39], pre-immunization with PM16 with adjuvant induced the formation of specific anti-phage antibodies capable of neutralizing phage particles and inhibited its lytic activity in vitro. However, the presence of anti-PM16 antibodies did not diminish the capacity of PM16 to boost the anti-*P. mirabilis* humoral response upon challenge, supporting the emerging opinion that neutralization in vitro does not directly predict therapy failure in vivo [1]. The most intriguing finding emerged from control groups: mice infected with *P. mirabilis* alone, in the absence of any phage immunization or therapy, developed serum antibodies that could bind and partially neutralize PM16 in vitro. This result complies with observations in other phage–host systems [40] and testifies to a clear paradox. It suggests that the bacterial infection itself presents antigenic stimuli cross-reactive with the phage particle.

To resolve this paradox, we hypothesized that *P. mirabilis* could produce proteins that share epitopes with lytic phages like PM16. Indeed, it was found that the key PM16 structural protein, particularly the receptor-binding spike protein gp45, is conserved across *Proteus* podophages and is enriched in B-cell epitopes predicted by Bepipred3 (Figure 6). Importantly, sequences encoding similar proteins were identified within the genomes of diverse *P. mirabilis* strains and non-related *Proteus* phages. This provides a possible genomic reservoir for the antigens driving the observed cross-reactive antibody response. The highly conserved structure of these proteins, especially spike proteins, indicates a strong evolutionary pressure, probably reflecting their critical function in interaction with the host, which makes them targets for a cross-reactive immune strategy.

We propose that bacteria can indirectly defend themselves against lytic phages by using the adaptive immune system of the macroorganism. The expression of conserved, prophage-derived structural proteins (like gp45) during a bacterial infection primes the macroorganism to produce antibodies against epitopes shared with lytic phages. This mechanism is a form of molecular mimicry between bacteria and phages, where the bacterium “vaccinates” a macroorganism against a viral predator, potentially providing a population-level benefit to lysogenic strains. This mechanism is similar to that which the bacteria can use for human-associated molecular mimicry described in a recent study [41]. In addition, the possibility of the formation of antibodies directed at phage receptors on the surface of host cells, which directly blocks phage infection, should be taken into account [42].

The paradigm that phage therapy efficacy depends on the macroorganism’s immune response has been stated by several authors [40,43]. The concern regarding phage-specific antibodies and their neutralization potential has been raised as one of the major questions in the upcoming era of phage therapy [44]. Moreover, it has also been shown that antibody-mediated phage neutralization does not necessarily abrogate phage treatment efficacy [45]. Our results regarding PM16 immune cross-reactivity with its bacterial target *P. mirabilis* are well explained by immune system engagement during different phage therapy regimens and align well with the idea that phage neutralization, while a legitimate concern, is not an absolute limit for phage therapy [30]. Additionally, our data demonstrate bacteria-mediated bacteriophage-specific immunization, which may result from the complex evolutionary interactions between different bacteriophages and bacteria, and may contribute to the production of phage-specific antibodies as a result of macroorganism interaction with bacteria.

It suggests that the patient’s pre-existing susceptibility or resistance to a therapeutic phage could be influenced by the patient’s history of infections with bacteria harboring specific prophages. For phage cocktail designs, it predicts that cross-neutralization may not only occur between administered phages but can also be pre-determined by the patient’s infection-induced antibody repertoire. In this scope, the screening of clinical bacterial isolates for their “phageome” as part of personalized phage selection could be useful for therapy.

The obtained data confirm a proof-of-concept of complex immune interactions that can occur as a result of phage therapy. However, it is important to note that this study was limited to a single phage–bacterium pair used for the animal model. Furthermore, while our genomic analysis is strongly supportive, direct experimental confirmation of the expression of these conserved proteins during infection is required. In this regard, it is premature to generalize our conclusions to any pair of phages and hosts, which requires further large-scale, comparative studies across a diverse panel of phages with different morphotypes and immunogenicity; however, this task is beyond the scope of the current investigation.

## 5. Conclusions

This study reveals that the humoral immune landscape of phage therapy is shaped by a complex, tripartite dialog between the therapeutic phage, the target bacterium, and the host immune system. We demonstrated that phage-neutralizing antibodies can emerge from the bacterial infection itself, a phenomenon explained by our discovery of highly conserved, potentially immunogenic phage proteins encoded within bacterial genomes. This leads us to propose the novel concept that bacteria can leverage the host’s adaptive immunity as an indirect defense against phages. Further investigation of these interactions will be crucial for advancing phage therapy from a largely empirical treatment to a predictable and precise component of infectious disease medicine.

## Figures and Tables

**Table 1:** Mixed linear effect model estimation on fluorescence intensity analysis of serological response to *P. mirabilis*.

Subgroup Pairs	Meandiff (log)	95% CI	*p*-Val
Subgroup 2 vs. Subgroup 3	0.0106	[0.0099, 0.0112]	<0.001
Subgroup 5 vs. Subgroup 6	0.0566	[0.0559, 0.0573]	<0.001
Subgroup 8 vs. Subgroup 9	0.0301	[0.0294, 0.0308]	<0.001
Subgroup 1 vs. Subgroup 4	−0.0038	[−0.0045, −0.0031]	<0.001
Subgroup 1 vs. Subgroup 7	−0.0009	[−0.0016, −0.0002]	0.0015
Subgroup 4 vs. Subgroup 6	0.1033	[0.1027, 0.104]	<0.001
Subgroup 4 vs. Subgroup 7	0.0029	[0.0022, 0.0036]	<0.001

**Table 2:** Mixed linear effect model estimation on serum-mediated prevention of PM16 adsorption on *P. mirabilis* cells.

Subgroup Pairs	Meandiff (log)	95% CI	*p*-Val
Subgroup 2 vs. subgroup 1	−0.0305	[−0.0307, −0.0303]	<0.001
Subgroup 3 vs. subgroup 2	−0.0031	[−0.0033, −0.0029]	<0.001
Subgroup 5 vs. subgroup 4	−0.0149	[−0.0151, −0.0147]	<0.001
Subgroup 6 vs. subgroup 5	−0.0062	[−0.0064, −0.0060]	<0.001
Subgroup 8 vs. subgroup 7	−0.0175	[−0.0177, −0.0173]	<0.001
Subgroup 9 vs. subgroup 8	−0.0045	[−0.0047, −0.0043]	<0.001

## Data Availability

The original contributions presented in this study are included in the article/Supplementary Material. Further inquiries can be directed to the corresponding authors.

## Supplementary Materials

The following supporting information can be downloaded at: https://www.mdpi.com/article/10.3390/v18060669/s1, Figure S1: IgG binding validation titration; File S1: IgG binding validation titration.

## Abbreviations

The following abbreviations are used in this manuscript:

UTIs	Urinary Tract Infections
CAUTIs	Catheter-Associated UTIs
IgA	Immunoglobulin A
CPSs	capsular polysaccharides
CFA	Complete Freund’s Adjuvant
IFA	Incomplete Freund’s Adjuvant
IgG	Immunoglobulin G
ELISA	Enzyme-Linked Immunosorbent Assay
PBS	Phosphate-Buffered Saline
LB	Lysogeny Broth
SD	Standard Deviation
MOI	Multiplicity Of Infection
PFU	Plaque-Forming Units
CFU	Colony-Forming Unit
